# PCK2 Inhibition Reverses Cisplatin Resistance of Non‐Small Cell Lung Cancer by Triggering Ferroptosis

**DOI:** 10.1096/fj.202602464R

**Published:** 2026-08-14

**Authors:** Jing Liu, Ziyan Wang, Chen Gu, Yili Chen, Yang Yang, Jianjie Zhu, Yihua Zhang, Yuna Shao, Xiaodong Pang, Yujie Ge, Huiwen Qian, Jian‐an Huang, Zeyi Liu, Yuanyuan Zeng, Cheng Ji

**Affiliations:** ^1^ Department of Pulmonary and Critical Care Medicine The First Affiliated Hospital of Soochow University Suzhou China; ^2^ Department of Respiratory Medicine Taixing Second People's Hospital Taixing China; ^3^ Institute of Respiratory Diseases Soochow University Suzhou China; ^4^ Department of Pharmacy The First Affiliated Hospital of Soochow University Suzhou China; ^5^ Department of Pulmonary and Critical Care Medicine, Lung Cancer Center The First Affiliated Hospital of Soochow University Suzhou China

**Keywords:** Cisplatin, Ferroptosis, NSCLC, PCK2, RGB‐286638 free base

## Abstract

Non‐small cell lung cancer (NSCLC) accounts for ~85% of lung cancers, with platinum‐based chemotherapy as the main treatment. Ferroptosis has been implicated in cancer chemoresistance, yet the molecular mechanisms linking metabolic reprogramming to ferroptosis‐mediated cisplatin resistance in NSCLC remain elusive. In this study, we found that phosphoenolpyruvate carboxykinase 2 (PCK2), a metabolic reprogramming enzyme, was significantly upregulated, and its overexpression enhanced ferroptosis resistance, thereby promoting cisplatin chemoresistance. Mechanistically, RGB‐286638 free base (RGB) inhibits PCK2 expression by directly binding to its R454 site to activate ferroptosis in cisplatin‐resistant cells and restores cisplatin sensitivity both in vitro and in vivo. Targeting PCK2 with RGB provides a promising strategy to overcome chemoresistance, facilitating improved clinical interventions for NSCLC.

## Graphical abstract

Metabolic enzyme PCK2 is aberrantly upregulated in cisplatin‐resistant NSCLC cells, which inhibits ferroptosis and ultimately facilitates tumor chemoresistance. The small‐molecule inhibitor RGB‐286638 directly binds to the key R454 functional site of PCK2 protein, efficiently inhibits PCK2 expression, and rescues PCK2‐mediated ferroptosis suppression. This pharmacological intervention restores cisplatin sensitivity of resistant NSCLC cells and suppresses tumor growth, providing a novel and actionable targeted strategy to overcome NSCLC chemotherapy resistance.

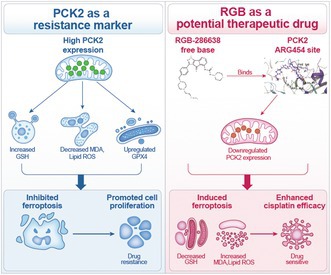

**Abbreviations**
CETSA: Cellular thermal shift assay
DARTS: Drug affinity responsive target stability
DDP: Cisplatin
FITC: Fluorescein isothiocyanate
FTH1: Ferritin heavy chain 1
GPX4: Glutathione peroxidase 4
GSH: Glutathione
HE: Hematoxylin–eosin
IHC: Immunohistochemistry
Lipid ROS: Lipid reactive oxygen species
MDA: Malondialdehyde
NSCLC: Non‐small cell lung cancer
PCK2: Phosphoenolpyruvate carboxykinase 2
qRT‐PCR: Quantitative real‐time reverse transcription PCR
RGB: RGB‐286638 free base
SLC7A11: Solute carrier family 7 member 11
SPR: Surface plasmon resonance
TEM: Transmission electron microscopy

## 1 Introduction

Chemotherapy remains a cornerstone in the clinical management of non‐small cell lung cancer (NSCLC), serving as a primary therapeutic strategy for inhibiting tumor progression and improving patient survival [1, 2, 3]. However, the acquisition of chemoresistance represents a major impediment to the efficacy of chemotherapeutic agents in NSCLC treatment, often leading to treatment failure, disease recurrence, and poor clinical outcomes. NSCLC cells develop chemoresistance through multifaceted mechanisms, including enhanced drug efflux, activation of anti‐apoptotic signaling pathways, dysregulation of DNA damage repair machinery, and remodeling of the tumor microenvironment [4, 5]. These adaptive alterations enable NSCLC cells to evade the cytotoxic effects of chemotherapeutics, posing a significant challenge to current NSCLC treatment paradigms.

Ferroptosis, a distinct form of regulated cell death characterized by iron‐dependent lipid peroxidation and the disruption of cellular redox homeostasis, has emerged as a promising therapeutic target to overcome tumor chemoresistance [6]. Unlike apoptosis, necrosis, or other cell death modalities, ferroptosis is driven by the accumulation of reactive oxygen species (ROS)‐mediated lipid peroxides, which are tightly regulated by a network of antioxidant defense systems, such as the glutathione (GSH)‐glutathione peroxidase 4 (GPX4) pathway, solute carrier family 7 member 11 (SLC7A11)‐mediated cystine import, and thioredoxin (TXN)‐thioredoxin reductase (TXNRD) system [7, 8, 9, 10, 11, 12].

In recent years, extensive studies have uncovered the intricate crosstalk between ferroptosis regulatory mechanisms and chemoresistance in NSCLC [13, 14, 15, 16]. Given the urgent need to address chemoresistance in clinical NSCLC oncology, understanding the molecular mechanisms that link ferroptosis to NSCLC chemoresistance has become a research focus. This study aims to elucidate phosphoenolpyruvate carboxykinase 2 (PCK2)‐mediated regulation of ferroptosis, thereby mediating resistance to chemotherapy, and discuss the potential strategy for enhancing the efficacy of chemotherapy in NSCLC. Such insights may lay the foundation for the development of novel combinatorial therapeutic regimens to overcome NSCLC chemoresistance and improve the clinical prognosis of NSCLC patients.

## 2 Materials and Methods

### 2.1 Cell Culture

The A549 and A549/cisplatin‐resistant cell lines (A549/DDP) were purchased from Procell Life Science & Technology Co. Ltd (Wuhan, China), and authenticated by the provider through comprehensive procedures, including mycoplasma contamination detection. A549 cells were cultured in RPMI 1640 medium (Gibco, Carlsbad, CA, USA), while the cisplatin‐resistant subline A549/DDP was maintained in A549/DDP‐specific medium (Procell, China). Both media were supplemented with 10% heat‐inactivated fetal bovine serum (FBS; Gibco, Carlsbad, CA, USA) and L‐glutamine (Invitrogen, Carlsbad, CA, USA). All cell cultures were incubated at 37°C in a humidified incubator with 5% CO_2_, and cells were passaged every 2–3 days to maintain the exponential growth phase.

### 2.2 Chemicals, Reagents and Antibodies

Cell Counting Kit‐8 (CCK‐8; APExBIO) was applied for detecting cell viability. Anti‐PCK2 (8565T), anti‐γ‐H2AX (9718S), anti‐BCL‐2 (15071S) antibodies were purchased from Cell Signaling Technology (Beverly, MA, USA). Anti‐BAX (50599‐2‐Ig), anti‐PCNA (60097‐1‐Ig), anti‐PCK2 (67676‐1‐Ig), anti‐SLC7A11 (26864‐1‐AP), anti‐GPX4 (67763‐1‐Ig), anti‐FTH1 (11682‐1‐AP) antibodies were obtained from Proteintech. RGB‐286638 free base and Neomycin Sulfate were purchased from TargetMol (Shanghai, China). Cisplatin (DDP), Necrostatin‐1, Z‐VAD‐FMK, Ferrostatin‐1, and Chloroquine were purchased from MedChemexpress (USA).

### 2.3 CCK‐8 Cell Viability Assay

Cells were seeded in 96‐well plates at a density of 3000 cells per well, with each well supplemented with 100 μL complete medium containing 10% FBS. Following 24 h of incubation for cell adhesion, cells were treated with various concentrations of DDP or RGB‐286638 free base (RGB) for 48 or 72 h, while untreated cells served as the control group. Absorbance values at 450 and 630 nm were detected using a microplate reader.

### 2.4 EdU

The EdU proliferation assay kit (Cat. No. C10310‐1) was purchased from RiboBio Co. Ltd. (Guangzhou, China). The assay was performed in strict accordance with the manufacturer's instructions. Fluorescent images were acquired using a Leica SP8 laser scanning confocal microscope (Leica Microsystems, Wetzlar, Germany) and analyzed with Leica Application Suite (LAS) software (Version X; Leica Microsystems).

### 2.5 Immunofluorescence Assay

Sterile cell slides were placed in 12‐well plates. Cells were trypsinized, centrifuged (1000 rpm, 5 min), resuspended, seeded, and incubated to 40%–50% confluence. Cells were washed with 500 μL PBS per well. Fixed with 200 μL 4% paraformaldehyde per well (15 min), washed with PBS, permeabilized with 200 μL 0.5% Triton X‐100/PBS (20 min), and washed with PBS again. Blocked with 200 μL 5% BSA per well (30–60 min), incubated with primary antibodies overnight at 4°C, then washed with 500 μL PBST per well (3 min each). Incubated with 200 μL 1:500 fluorescent secondary antibodies (1 h, in the dark), washed with 500 μL TBST per well (3 min each). Stained with 200 μL 1:1000 DAPI/PBS (3 min, in the dark), washed with PBST. Slides were mounted cell‐side down on anti‐quenching agent‐coated glass slides, stored in the dark, and imaged by confocal microscopy.

### 2.6 Western Blot

Cells in 6‐well plates were washed twice with PBS and lysed on ice with 2 × SDS lysis buffer. Lysates were transferred to EP tubes, vortexed, briefly centrifuged, and boiled at 100°C for 7 min. Proteins were separated by SDS‐PAGE using 10% or 12.5% gels (80 V for stacking, then 140 V for resolving), followed by transfer onto nitrocellulose membranes at 250 mA for 2 h at 4°C. Membranes were stained with Ponceau S to confirm transfer, blocked for 1 h at room temperature, and incubated with primary antibodies overnight at 4°C. After washing with TBST, membranes were incubated with HRP‐conjugated secondary antibodies for 2 h at room temperature. Protein bands were visualized using ECL chemiluminescence.

### 2.7 Quantitative Real‐Time Reverse Transcription PCR (qRT‐PCR)

Total RNA was extracted from cells grown to ~90% confluence in 6‐well plates using 1 mL of ice‐cold Trizol per well. After adding 200 μL of chloroform, the mixture was centrifuged at 12000 rpm for 15 min at 4°C. The aqueous phase was transferred to a new RNase‐free tube, mixed with an equal volume of isopropanol, and centrifuged again. The RNA pellet was washed twice with 75% ethanol, air‐dried, and dissolved in 20 μL of DEPC water. For cDNA synthesis, RNA (2 μL) was mixed with Random Primer (1 μL), dNTP (1 μL), and DEPC water (6 μL), heated at 65°C for 5 min, and chilled on ice. Subsequently, 5 × RTase Reaction Buffer (4 μL), DEPC water (5.5 μL), and M‐MLV reverse transcriptase (0.5 μL) were added. Reverse transcription was performed at 30°C for 10 min, 42°C for 60 min, and 70°C for 15 min. qRT‐PCR was carried out using SYBR Green Master Mix with specific primers for PCK2, SLC7A11, GPX4, FTH1, and β‐actin. The cycling conditions were 95°C for 10 min, followed by 40 cycles of 95°C for 15 s and 60°C for 1 min. Relative gene expression was calculated using the 2^−ΔΔCt^ method. The primer sequences used for qRT‐PCR were as follows: primer sequences for PCK2 (sense, 5′‐TCCCACTGGCATTCGAGATT‐3′; anti‐sense, 5′‐CCGTCTTGTCTCTCTACTGCT‐3′), primer sequences for SLC7A11 (sense, 5′‐TTACCAGCTTTTGTACGAGTCT‐3′; anti‐sense, 5′‐GTGAGCTTGCAAAAGGTTAAGA‐3′), primer sequences for GPX4 (sense, 5′‐GGAGUAACGAAGAGAUCAA‐3′; anti‐sense, 5′‐UUGAUCUCUUCGUUACUCC‐3′), primer sequences for FTH1 (sense, 5′‐CTCCTACGTTTACCTGTCCATG‐3′; anti‐sense, 5′‐CAAGTCAATCAGGCACATACAAG‐3′), and primer sequences for β‐actin (sense, 5′‐CCAACCGCGAGAAGATGACC‐3′; anti‐sense, 5′‐AGGAAGGCTGGAAAAGAGCC‐3′).

### 2.8 Glutathione (GSH) Assay

Total GSH levels were determined using a GSH assay kit (Beyotime, Shanghai, China). A549 and A549/DDP cells were seeded in 6‐well plates at a density of 2 × 10^5^ cells per well. After cell attachment, the cells were treated with different concentrations of DDP or RGB for 48 h. Subsequently, the cells were harvested, resuspended in PBS, and lysed by sonication. The supernatant was collected by centrifugation for subsequent GSH level determination.

### 2.9 Malondialdehyde (MDA) Assay

Total MDA levels were measured using an MDA assay kit (Solarbio, Beijing, China). A549 and A549/DDP cells were seeded in 6‐well plates at a density of 2 × 10^5^ cells per well. Following treatment with DDP or RGB for 48 h, the cells were harvested, resuspended in PBS, and lysed by sonication. The supernatant was obtained by centrifugation for the detection and analysis of MDA levels.

### 2.10 Detection of Ferrous Iron (Fe^2+^) by FerroOrange Staining

A549 and A549/DDP cells were seeded in confocal dishes at a density of 1 × 10^5^ cells per dish and treated with DDP or RGB for 48 h. After removing the medium, the cells were incubated with serum‐free medium containing 1 μM FerroOrange probe (Dojindo China Co. Ltd., Shanghai, China) at 37°C in the dark for 30 min. The cells were washed three times with PBS, and fluorescence images were acquired using a laser confocal microscope. The fluorescence intensity was quantitatively analyzed using ImageJ software.

### 2.11 Quantification of Intracellular Lipid Reactive Oxygen Species (ROS) Levels

After A549 and A549/DDP cells were treated with DDP or RGB for 48 h, the cells were collected and centrifuged at 1000 rpm for 3 min. The supernatant was discarded, and the cell pellet was gently resuspended in 500 μL of medium containing 0.02% ROS probe, followed by incubation at 37°C in the dark for 30 min. After centrifugation and removal of the supernatant, the cell pellet was resuspended in 200 μL of PBS. Intracellular ROS levels were quantified by flow cytometry.

### 2.12 Transfection Assay

Cells were digested and seeded into 6‐well plates, incubated to reach 50%–60% confluence. After PBS washing, serum‐free medium was added. siRNA and Lipofectamine 2000 were separately diluted in serum‐free medium (Tube A and Tube B) and equilibrated for 5 min at room temperature. The two diluents were combined and incubated 10 min to form transfection complexes. A total of 500 μL complex mixture was added to each well, followed by gentle plate rocking and 6 h incubation; medium was refreshed promptly if abnormal cell morphology occurred. After rinsing with PBS, complete medium was replenished, and cells were cultured for another 48 h before downstream detection. The interfering RNAs, gene knockdown and overexpression viruses used in this study were all synthesized by Suzhou GenePharma Co. Ltd., with the nucleotide sequences listed as follows: negative control (NC): TTCTCCGAACGTGTCACGT; si‐PCK2‐1/sh‐PCK2‐1: GCGAGTGCTTAGTGGAGATCT; si‐PCK2‐2/sh‐PCK2‐2: GGTGGCAAGCATGCGTATTAT.

### 2.13 Lentiviral Transduction and Stable Cell Line Selection

Lentiviral vectors (shNC, shPCK2‐1, shPCK2‐2) were synthesized by GenePharma. The nucleotide sequences of the lentiviral constructs were the same as above. Target cells were seeded into 6‐well plates at 3 × 10^4^–5 × 10^4^ cells/mL. When cells reached 40%–50% confluence, they were washed twice with PBS and transduced with 1 mL of lentiviral supernatant, 1 mL of complete medium, and 1.6 μL of polybrene (at a final concentration of 0.8 μL/mL). After 48 h, transduction efficiency was assessed by fluorescence microscopy. Cells were then passaged into culture flasks and selected with puromycin starting at 0.5 μg/mL, with stepwise increases up to 4 μg/mL as needed. Selection continued until floating cells were no longer observed and ≥ 95% of cells were fluorescence‐positive. Stable cell lines were then harvested for validation of target gene knockdown or overexpression by qRT‐PCR and Western blot.

### 2.14 Transmission Electron Microscopy (TEM)

TEM analysis was performed by Aifang Biotechnology. A549/DDP shNC and A549/DDP shPCK2 cells were cultured to the logarithmic growth phase (≥ 60% confluence), treated with 5 μM DDP for 48 h, and harvested with at least 3 × 10^6^ cells. Cells were fixed with 2.5% glutaraldehyde for 5 min at room temperature in the dark, then gently scraped in a single direction using a cell scraper or a soft rubber blade to minimize cell damage. The cell suspension was transferred to a centrifuge tube and centrifuged at ≤ 3000 rpm for approximately 2 min to obtain a cell pellet the size of a mung bean. The fixative was replaced with fresh electron microscopy‐specific fixative, and the cell pellet was gently resuspended. After fixation for 30 min at room temperature in the dark, samples were stored at 4°C and transported with ice packs, avoiding freezing.

### 2.15 FITC Labeling of RGB‐286638 Free Base

Fluorescein isothiocyanate (FITC) was used for fluorescence labeling via amino groups. RGB powder was dissolved in DMSO, and the pH was adjusted to 6.0 with hydrochloric acid. Hydroxylamine hydrochloride was added, followed by EDC/NHS and 5(6)‐aminofluorescein. The mixture was allowed to react for 24 h in the dark to obtain the RGB‐FITC conjugate. The labeled product was characterized by full‐wavelength scanning (FITC excitation: 492 nm; emission: 520 nm).

### 2.16 Molecular Docking

The crystal structure of PCK2 was predicted using AlphaFold3 and imported into Schrödinger. Protein preparation was performed using the Protein Preparation Wizard, including preprocessing, hydrogen bond assignment, energy minimization, and water removal. The 2D structure of RGB was processed with the LigPrep module to generate all possible 3D chiral conformations. Potential binding sites on PCK2 were predicted using the SiteMap module, and an enclosing box was generated with the Receptor Grid Generation module. Molecular docking was performed using extra precision (XP) mode. Binding stability was further evaluated using MM‐GBSA (molecular mechanics with general Born and surface area) binding free energy calculations, where lower MM‐GBSA dG Bind values indicate more stable ligand‐protein interactions.

### 2.17 Drug Affinity Responsive Target Stability (DARTS)

Cellular extracts were prepared using a buffer containing 0.4% Triton X‐100, 400 mM NaCl, 100 mM Tris–HCl (pH 7.5), and 20% glycerol. The protein concentration in the extracts was determined by the BCA assay (Beyotime Biotechnology, China). Extracts were mixed with RGB or DMSO and incubated at 4°C overnight. On the second day, pronase was added to the samples, followed by incubation for 20 min. Finally, the reaction was terminated by adding 5 × loading buffer, and the mixture was subjected to Western blot analysis.

### 2.18 Cellular Thermal Shift Assay (CETSA)

To extract total proteins, A549/DDP cells with different treatments were lysed on ice for 30 min. The samples were treated with RGB or DMSO at room temperature for 60 min. Subsequently, the samples were transferred to PCR tubes and heated at 10 different temperatures for 5 min each. After heating, the samples were kept at 4°C for 3 min, centrifuged at 12000*g* for 10 min, and mixed with 2× buffer. Finally, the samples were heated at 95°C for 7 min and then detected by western blot.

### 2.19 Surface Plasmon Resonance (SPR)

The interaction between the PCK2 and RGB was quantified using a Biacore 1K (Cytiva, Sweden) with a CM5 sensor chip. The PCK2 protein (50 μg/mL) was adjusted to pH 4.0 and then immobilized on the surface of the chip cell at 10 μL/min for 5 min. The chip cell was then blocked with 1 M ethanolamine (10 μL/min for 10 min). A neighboring cell that served as a reference was similarly activated and blocked, except that PBS adjusted to pH 4.0 was used for immobilization. RGB was diluted to six concentrations in PBS + 5% DMSO. The flow rate was set to 30 μL/min for 150 s in each run, which includes a combination of 60 s and a dissociation of 90 s. Biacore Insight Evaluation (v4.0.8.20368) was used to calculate the kinetic parameters of KD.

### 2.20 Patient‐Derived Organoid (PDO)

Fresh LUAD PDO tumor tissues were minced, washed with ice‐cold PBS, and then digested with EDTA. After Matrigel polymerization at 37°C for 10 min, advanced DMEM/F12 was supplemented with penicillin/streptomycin, 2 mM GlutaMAX, 10 mM HEPES, 1 × B27, 1 × N2 (Life Technologies), 1 mM N‐acetylcysteine (Sigma), and 10 nM gastrin I (Biogems). The following factors were also added: 50 ng/mL recombinant EGF, 500 nM A83‐01 (Biogems), 100 ng/mL recombinant Noggin (Peprotech), 10 μM Y‐27632 (Abmole), 500 ng/mL R‐spondin‐1 (Peprotech), 10 mM nicotinamide (Sigma), and 10 μM SB202190 (Sigma). Once organoids formed, the original medium was removed, and organoids were treated with 1 μM RGB. After RGB treatment, 4 PDOs of similar sizes were selected for observation. These organoids were imaged on Day 7, and their sizes were measured using ImageJ. All samples were collected with patients' written informed consent.

### 2.21 In Vivo Tumorigenesis Assay

Four‐week‐old female nude mice were randomized into equal groups, with 100 μL tumor cell suspension (4 × 10^6^ cells) subcutaneously inoculated into the right axilla of each mouse. Mice were monitored regularly for tumor formation and health status; successful inoculation was defined as visible subcutaneous tumor growth. Tumor volume was measured every 3 days with a vernier caliper and calculated using the formula V = L × D^2^/2 (L = longest diameter, D = shortest diameter). When tumors reached 100 mm^3^, intraperitoneal administration was performed every 3 days, with continuous monitoring of mouse health and tumor volume. At the end of the experiment, nude mice were euthanized humanely by cervical dislocation. Tumor status was recorded and photographed. Tumors were then measured, dissected, weighed, and processed for immunohistochemistry (IHC) and hematoxylin–eosin (HE) staining.

### 2.22 Statistical Analysis

All experimental data were analyzed using statistical methods and further processed with GraphPad Prism 8.0 software. Data are presented as mean ± standard deviation (mean ± SD), and each experiment was repeated at least three times. A *p*‐value < 0.05 was considered statistically significant (ns, no significant, *p < 0.05, **p < 0.01, ***p < 0.001).

## 3 Results

### 3.1 Ferroptosis Resistance Was Involved in Chemoresistant NSCLC Cells

**FIGURE 1:**
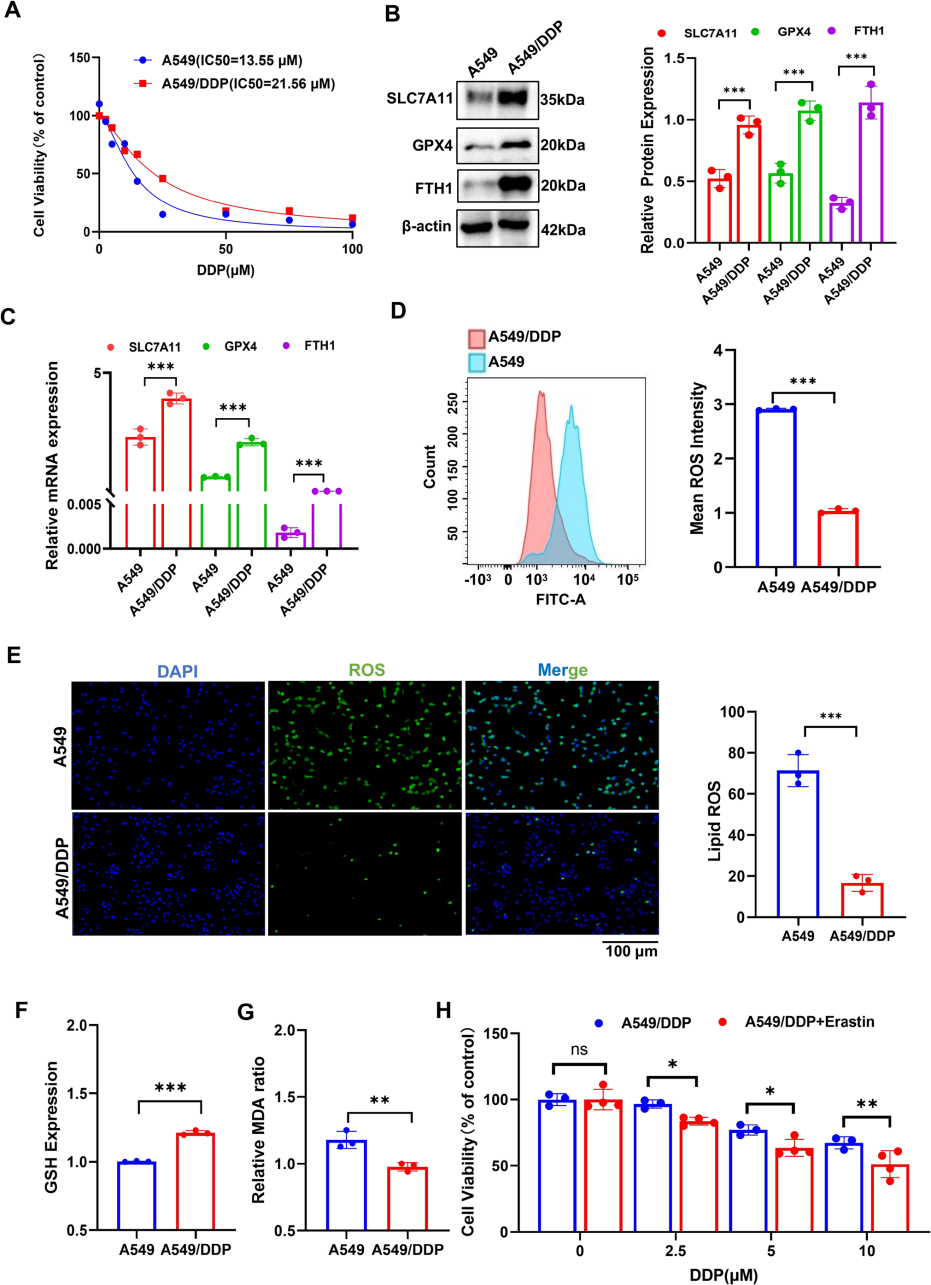
Detection of ferroptosis‐related pathway in chemoresistant NSCLC cells. (A) Detection of IC_50_ in A549 and A549/DDP cell lines. (B) Western blot analysis of the expression levels of SLC7A11, GPX4, and FTH1, in A549 and A549/DDP. (C) qRT‐PCR for SLC7A11, GPX4, and FTH1 mRNA levels in A549 and A549/DDP. (D) Flow cytometry analysis of Lipid ROS levels in A549 cells and A549/DDP. (E) Verification of Lipid ROS fluorescence intensity in A549 and A549/DDP. (F) Detection of GSH levels in A549 and A549/DDP. (G) Detection of MDA levels in A549 and A549/DDP. (H) CCK‐8 assay for the sensitivity of A549/DDP cells to cisplatin after Erastin treatment. (ns, no significant, *p < 0.05, **p < 0.01, ***p < 0.001).

We validated the half‐maximal inhibitory concentration (IC_50_) of DDP in parental A549 cells and DDP‐resistant A549/DDP cells (Figure 1A). Results indicated that the expression levels of key ferroptosis‐regulating proteins—including SLC7A11, GPX4, and FTH1—were significantly elevated in A549/DDP cells relative to parental A549 cells (Figure 1B). Consistently, qRT‐PCR assays demonstrated that the mRNA expression levels of SLC7A11, GPX4, and FTH1 were also significantly upregulated in A549/DDP cells (Figure 1C).

To further characterize the ferroptosis status of these cells, we assessed Lipid ROS levels via flow cytometry. Lipid ROS levels were significantly lower in A549/DDP cells compared to A549 cells (Figure 1D). This observation was further corroborated by staining with a specific Lipid ROS fluorescent probe: inverted fluorescence microscopy revealed notably diminished green fluorescence (indicative of the oxidized state) in A549/DDP cells relative to parental cells (Figure 1E). Analysis revealed that intracellular levels of GSH—a critical antioxidant—were significantly higher in A549/DDP cells than in A549 cells (Figure 1F). Conversely, the content of MDA, a well‐recognized marker of lipid peroxidation, was markedly reduced in A549/DDP cells (Figure 1G). These data collectively confirm that the extent of lipid peroxidation is significantly attenuated in DDP‐resistant cells. To explore the therapeutic potential of targeting ferroptosis in reversing DDP resistance, we treated A549/DDP cells with the ferroptosis inducer Erastin (10 μM) for 24 h, followed by cell viability assessment using the CCK‐8 assay. Results showed that Erastin treatment significantly reduced the survival rate of A549/DDP cells compared to the control group (Figure 1H), indicating that targeting the ferroptosis pathway can effectively reverse DDP resistance.

To explore the regulatory mechanisms underlying chemoresistance in tumor cells, we detected GSH after treatment with different concentrations of DDP (0, 5, and 10 μM). The GSH content in A549 cells was significantly increased in a dose‐dependent manner (Figure S1A), while the level of MDA showed a relative decrease (Figure S1B). We also evaluated the changes in Lipid ROS levels in A549 cells treated with different concentrations of DDP for 48 h using flow cytometry. Compared with the control group, the intracellular Lipid ROS levels in the DDP‐treated groups exhibited a significant dose‐dependent reduction (Figure S1C). Observations under an inverted fluorescence microscope revealed that the green fluorescent signal was notably attenuated with the increase of DDP concentration (Figure S1D), indicating that DDP can effectively inhibit intracellular lipid peroxidation. Additionally, through FerroOrange probe staining, we found that the intracellular Fe^2+^ content was also decreased in a drug concentration‐dependent manner (Figure S1E). In A549 and H1299 cell lines, treatment with gradient concentrations of DDP led to a decrease in expression of BCL‐2 and an increase of BAX. Concurrently, the expression of GPX4 was upregulated in both cell lines (Figure S1F,G). These results suggest that ferroptosis resistance is activated during DDP‐induced cellular apoptosis, which may serve as one of the important mechanisms underlying chemoresistance in tumor cells. Collectively, these findings suggest that NSCLC cells acquire DDP resistance through mechanisms including upregulating GSH synthesis, reducing iron ion accumulation, and inhibiting lipid peroxidation.

### 3.2 PCK2 Was Significantly Upregulated in Chemoresistant NSCLC Cells and Tissues

**FIGURE 2:**
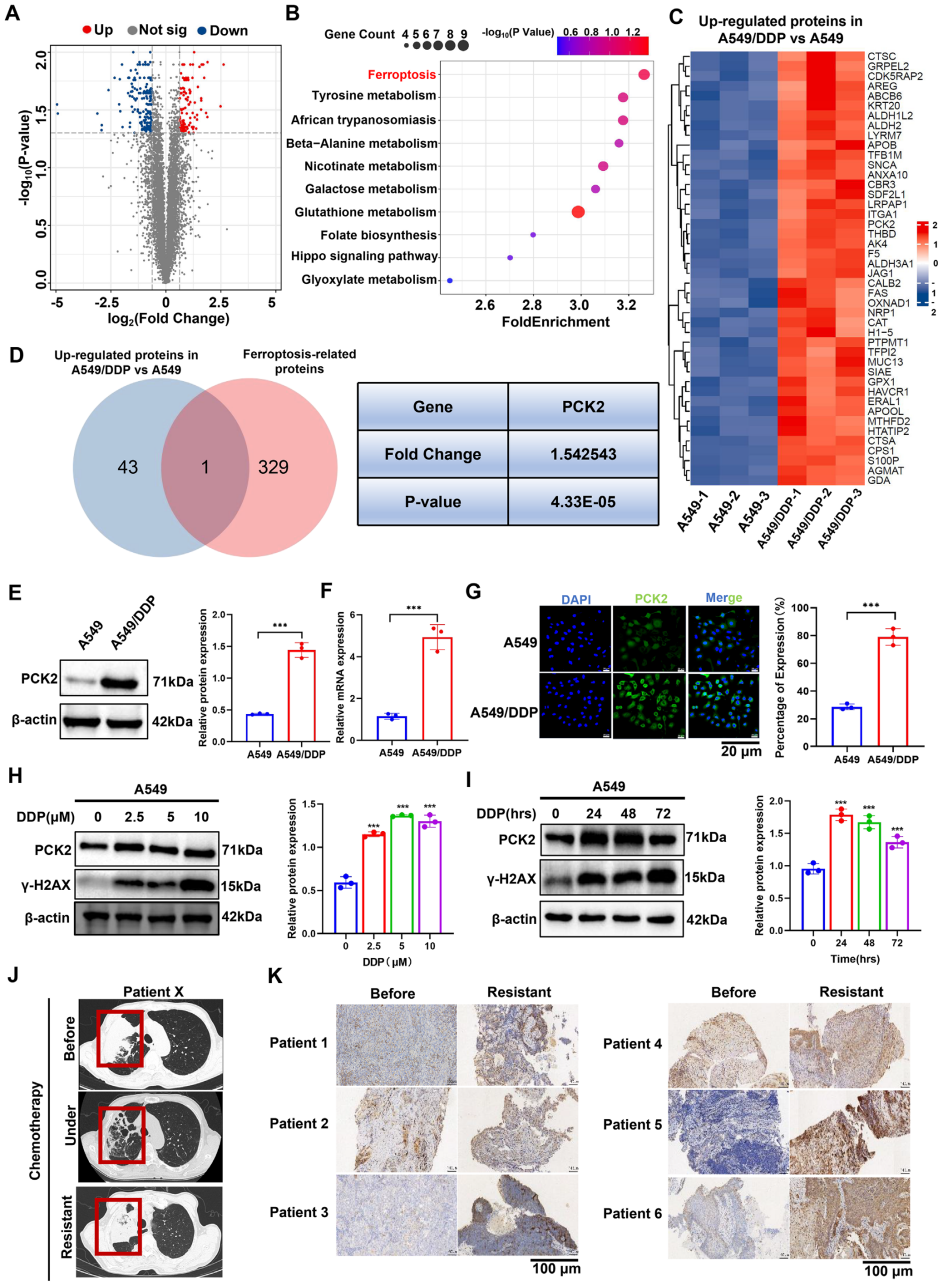
Expression of PCK2 in chemoresistant NSCLC Cells. (A) Differential protein volcano plot. (B) Differential protein heatmap. (C) GESA analysis. (D) Target proteins from upregulated differential proteins and ferroptosis gene intersection. (E) Protein expression level of PCK2 in A549 and A549/DDP cells. (F) mRNA expression level of PCK2 in A549 and A549/DDP cells. (G) Immunofluorescence expression of PCK2 in A549 and A549/DDP cells. (H) Western blot analysis of PCK2 protein expression in A549 cells treated with gradient concentrations of DDP (0, 2.5, 5, 10 μM) for 48 h. (I) Western blot analysis of PCK2 protein expression in A549 cells treated with gradient concentrations of time points (0, 24, 48, 72 h). (J) Representative CT images of NSCLC patients before chemotherapy, during chemotherapy, and after the development of drug resistance. (K) PCK2 in NSCLC patient specimens (chemotherapy sensitive vs. Chemotherapy resistant). (***p < 0.001).

We performed proteomic analysis on A549 cells and A549/DDP cells using high‐resolution mass spectrometry. Differentially expressed proteins identified by sequencing were screened with the following criteria: Fold Change > 1.2 and *p*‐value < 0.05. Volcano plot analysis revealed a total of 113 significantly upregulated proteins and 175 downregulated proteins (Figure 2A).

To further explore the biological functions of these proteins, we conducted Gene Set Enrichment Analysis (GSEA) on the significantly upregulated proteins. The results demonstrated that the upregulated proteins were closely associated with key biological processes in tumor cells, including ferroptosis and glutathione metabolism (Figure 2B).

To further screen proteins significantly correlated with ferroptosis, we applied stricter thresholds: Fold Change > 1.5 and *p*‐value < 0.0005, yielding 44 upregulated proteins (Figure 2C). A ferroptosis‐related gene set was downloaded from the FerrDb database (ferrdb‐zhounan.org). After merging and removing duplicates, 330 ferroptosis‐associated proteins were obtained. The intersection of these 44 differentially expressed proteins and the 330 ferroptosis‐related genes yielded one overlapping protein: PCK2 (Figure 2D).

To clarify the expression of PCK2 in chemoresistant NSCLC, we detected PCK2 expression at the protein, mRNA, and immunofluorescence levels in the A549/DDP cell line in vitro, which showed a significant upregulation compared to parental A549 cells (Figure 2E–G). Additionally, A549 cells were exposed to different concentrations of DDP for 48 h or treated with 5 μM DDP at various time points to assess PCK2 expression. The results demonstrated that DDP treatment induced a concentration‐dependent and time‐dependent increase in PCK2 protein expression (Figure 2H,I).

Furthermore, we collected lung tissue samples from NSCLC patients before and after clinical chemotherapy resistance (Figure 2J). Immunohistochemical (IHC) staining showed that PCK2 expression was significantly higher in post‐chemoresistance tissues compared to pre‐chemoresistance samples (Figure 2K). These findings confirm that PCK2 is overexpressed in chemoresistant cells and further suggest that PCK2 may be involved in promoting chemoresistance in NSCLC.

### 3.3 PCK2 Regulated Cisplatin Sensitivity of NSCLC Cells in vitro

**FIGURE 3:**
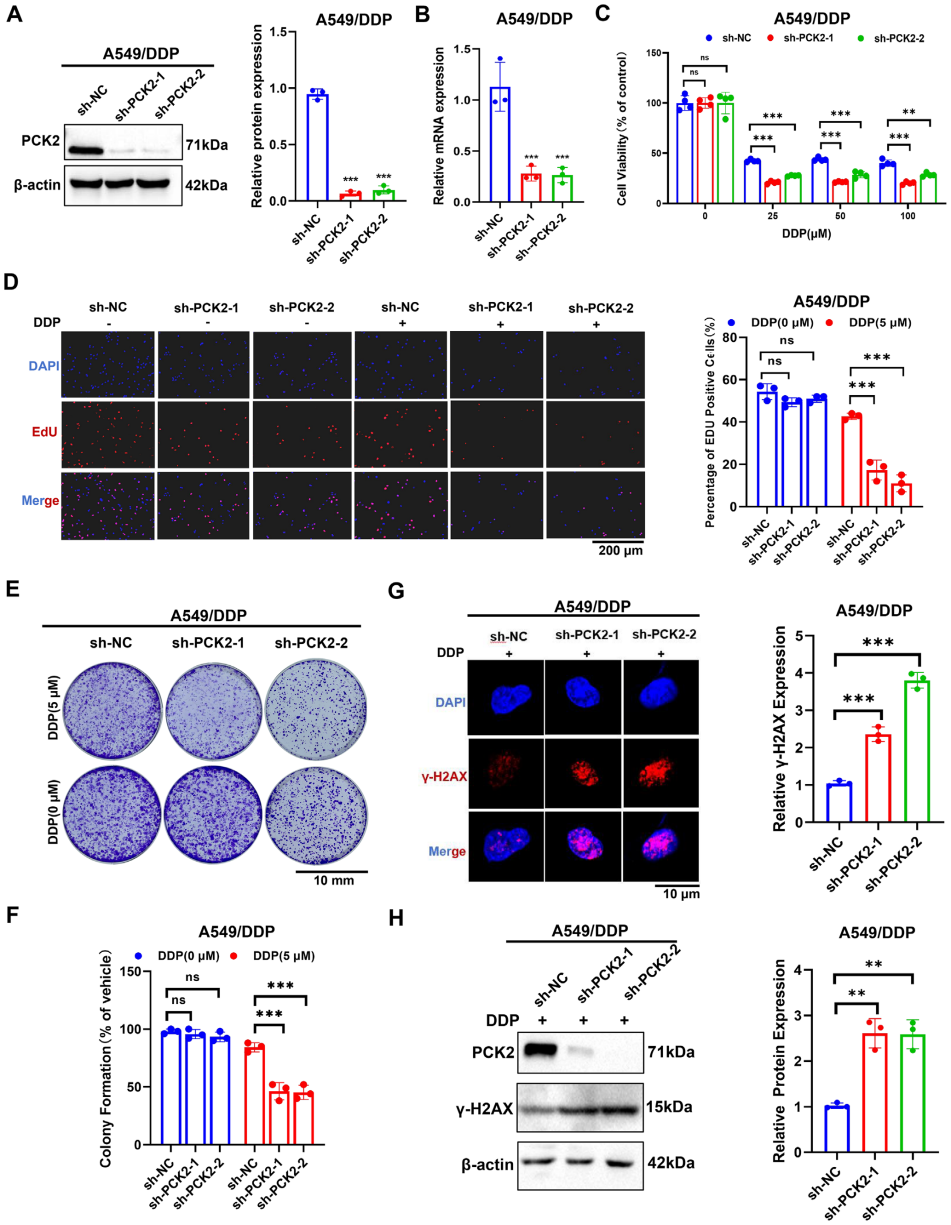
Knockdown of PCK2 enhances sensitivity of NSCLC to DDP. (A) Protein expression in shPCK2 cells. (B) mRNA expression in the shPCK2 cells. (C) Cell viability under treatment DDP. (D) Proliferative capacity of cells with EdU. (E, F) Colony formation assay. (G) Fluorescence expression of γ‐H2AX induced by PCK2 knockdown under DDP treatment. (H) Protein level of γ‐H2AX after PCK2 knockdown under DDP treatment. (ns, no significant, **p < 0.01, ***p < 0.001).

To investigate the role of PCK2 in chemoresistance of NSCLC, we successfully established a PCK2 stable knockdown cell line (shPCK2) in A549/DDP cells. Western blot and qRT‐PCR results confirmed that shPCK2 significantly downregulated PCK2 expression at both the protein and mRNA levels (Figure 3A,B). We evaluated the effects of PCK2 knockdown on cellular drug sensitivity and proliferative capacity through multi‐dimensional experiments. Firstly, CCK‐8 cell viability assay showed that under gradient concentrations of DDP treatment, the survival rate of shPCK2‐transfected cells was significantly lower than that of the control group (Figure 3C). EdU cell proliferation assay confirmed that the proliferation of shPCK2 cells was markedly inhibited following DDP exposure (Figure 3D). Consistently, colony formation assay showed that the number of colony formation in shPCK2 group was significantly reduced under DDP treatment (Figure 3E,F). These data indicate that PCK2 plays a crucial role in maintaining the proliferative capacity of DDP‐resistant cells. To explore the role of PCK2 in DNA damage response, we detected the expression change of γ‐H2AX using Western blot and immunofluorescence techniques. Both results demonstrated that the protein level of γ‐H2AX in the shPCK2 group was significantly higher than that in the control group after DDP treatment (Figure 3G,H). These results indicate that PCK2 may play a crucial protective role in resisting DDP‐induced cytotoxicity by regulating the DNA damage repair pathway, and its depletion can significantly enhance DDP‐triggered DNA damage effects.

To further validate the functional role of PCK2 in chemoresistance, we established PCK2‐overexpressing stable cell lines (OE‐PCK2) in A549 cells. Western blot and qRT‐PCR analysis demonstrated that both protein and mRNA levels of PCK2 were significantly upregulated in the OE‐PCK2 group compared with the control group (Figure S2A,B). CCK‐8 assays revealed that OE‐PCK2 cells exhibited a marked increase in cell viability following treatment with gradient concentrations of DDP (Figure S2C). Additionally, colony formation and EdU proliferation assays confirmed that PCK2 overexpression significantly enhanced the proliferative capacity of A549 cells under DDP treatment (Figure S2D,E). The impact of PCK2 overexpression on DDP‐induced DNA damage was evaluated using Western blot and immunofluorescence staining. Immunofluorescence analysis showed a notable reduction in γ‐H2AX expression in A549 OE‐PCK2 group, which was further corroborated by western blot results indicating that PCK2 overexpression significantly decreased γ‐H2AX protein levels in DDP‐treated relative to the control group (Figure S2F,G). Collectively, these findings suggest that PCK2 plays a critical protective role against DDP‐induced genotoxicity by enhancing cell proliferation, drug tolerance, and the capacity for DNA damage repair.

### 3.4 Overexpression of PCK2 Promoted DDP Resistance of NSCLC Cells in vivo

**FIGURE 4:**
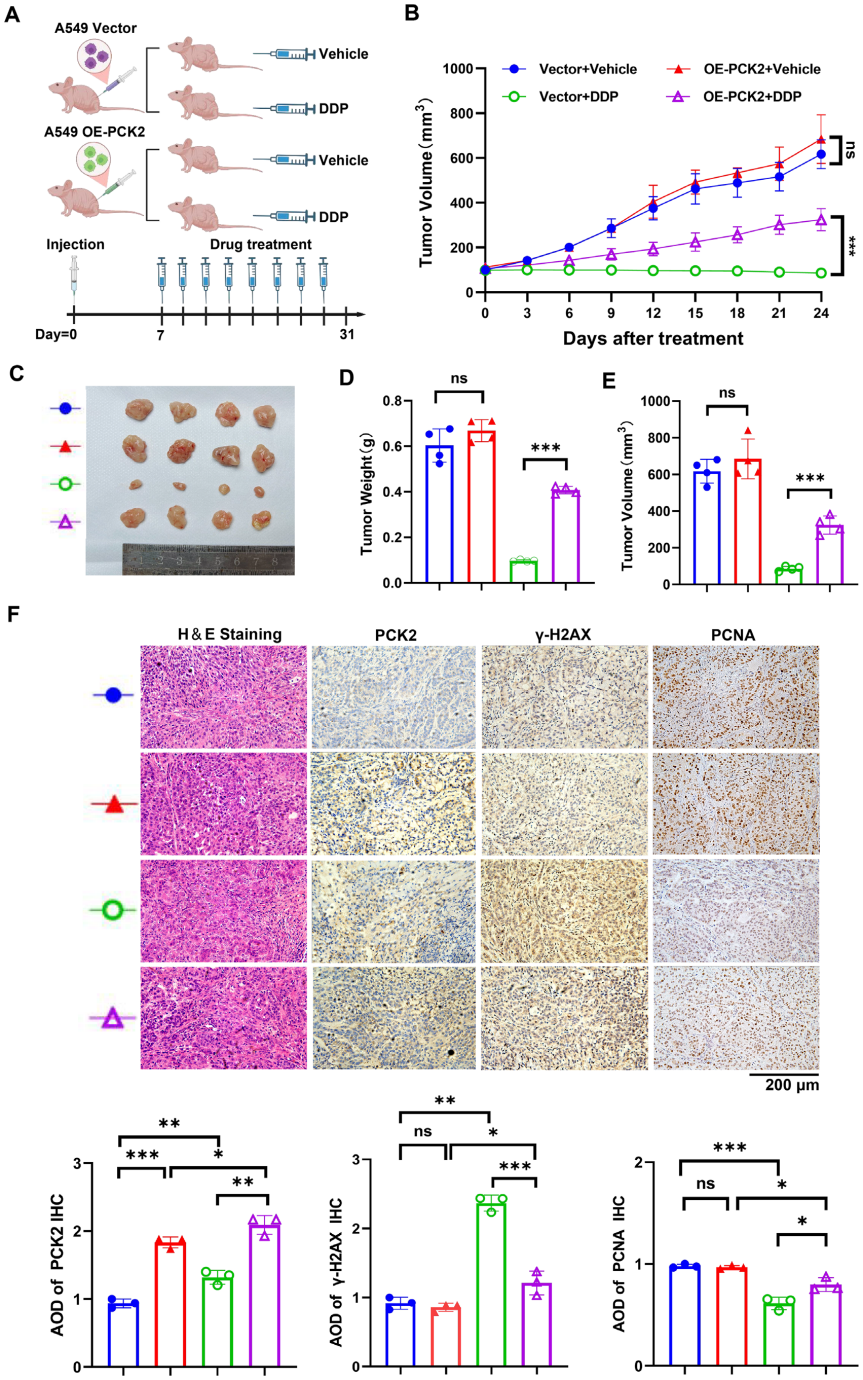
PCK2 attenuates the sensitivity of tumor cells to DDP in vivo. (A) Diagram showing the process of the experiments in vivo. (B) Effect of PCK2 on tumor. (C) Growth curves of xenograft tumors in vivo. (D) Tumor weight. (E) Tumor volume. (F) HE and IHC analysis of tumor tissues. (ns, no significant, *p < 0.05, **p < 0.01, ***p < 0.001).

In vivo validation experiments, sixteen 4‐week‐old female nude mice were randomly divided into four groups (*n* = 4 per group): Vector + Vehicle, OE‐PCK2 + Vehicle, Vector + DDP, and OE‐PCK2 + DDP (Figure 4A). Each mouse was subcutaneously inoculated with 100 μL of the corresponding tumor cell mixture (4 × 10^6^ cells). Following successful inoculation, the mice were monitored regularly for subcutaneous tumor formation and general health status. When tumors reached an approximate volume of 100 mm^3^, mice in each group were intraperitoneally injected with 5 mg/kg DDP or PBS every 3 days. At the end of the experiment, the mice were euthanized humanely, with relevant data recorded and tumors photographed (Figure 4B). During the treatment period, the general health status of the mice was closely observed, and tumor volumes were continuously measured (Figure 4C). Subsequent measurements of tumor volume were performed, followed by careful dissection and weighing of the tumor tissues (Figure 4D,E). We observed that PCK2 overexpression exerted no significant effect on the in vivo proliferative capacity of A549 cells under vehicle treatment; in contrast, upon DDP exposure, PCK2 overexpression markedly abrogated the tumor‐suppressive effect of DDP. Consistent with the aforementioned findings, IHC results further confirmed that PCK2 overexpression significantly reduced γ‐H2AX expression in A549 cells following treatment with DDP at the same concentration (Figure 4F). Our results demonstrated that PCK2 overexpression in A549 cells did not affect in vivo cell proliferation under vehicle treatment; however, under DDP exposure, PCK2 overexpression significantly promoted tumor growth. Both in vitro and in vivo experiments confirmed that elevated PCK2 expression contributes to the development of DDP resistance in A549 cells.

### 3.5 PCK2 Confers Cisplatin Chemoresistance by Triggering Ferroptosis Inhibition

**FIGURE 5:**
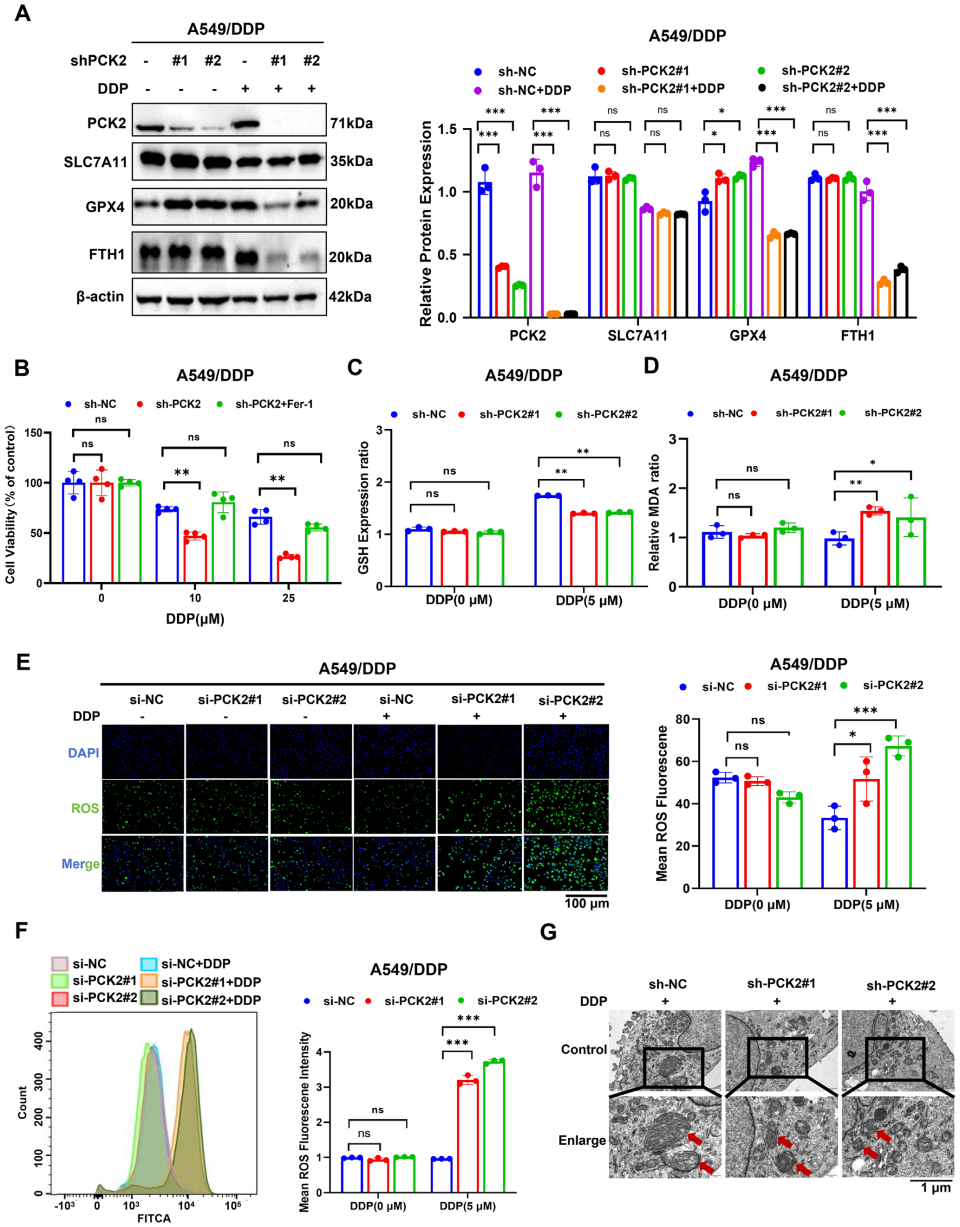
PCK2 knockdown promotes ferroptosis. (A) Expression of ferroptosis‐related proteins in A549/DDP cells transfected with shPCK2 or shNC, with or without DDP treatment. (B) CCK‐8 assay for cell proliferation activity in A549/DDP shPCK2 cells following treatment with the ferroptosis inhibitor Ferrostatin‐1 (Fer‐1). (C) GSH in A549/DDP shPCK2 cells treated with or without DDP. (D) MDA in A549/DDP shPCK2 cells treated with or without DDP. (E) Lipid ROS fluorescence intensity in A549/DDP cells transfected with siPCK2. (F) Lipid ROS in A549/DDP siPCK2 cells. (G) Mitochondrial and cellular morphological changes in A549/DDP shPCK2 cells observed under transmission electron microscopy (TEM) after 48 h of DDP treatment. (ns, no significant, *p < 0.05, **p < 0.01, ***p < 0.001).

Following treatment of A549/DDP cells with 5 μM DDP, protein expression levels of GPX4 and FTH1 in the shPCK2 group were significantly downregulated compared with the control group (Figure 5A). After PCK2 silencing in A549/DDP, cell viability was significantly reduced in a DDP dose‐dependent manner relative to the control group. Notably, intervention with the ferroptosis inhibitor Ferrostatin‐1 (Fer‐1) partially reversed this increase in drug sensitivity (Figure 5B). Concomitantly, GSH content was markedly decreased, while MDA levels were significantly elevated (Figure 5C,D). These findings further confirm that PCK2 may promote chemoresistance by mediating ferroptosis resistance. Fluorescence staining revealed that after 48 h of 5 μM DDP treatment, PCK2‐silenced (siPCK2) cells exhibited a significant upregulation of Lipid ROS levels compared with the control group (Figure 5E). This observation was further corroborated by flow cytometry, which demonstrated a marked increase in Lipid ROS production in A549/DDP siPCK2 cells following 48 h of DDP exposure (Figure 5F). Additionally, significant mitochondrial morphological alterations, including enhanced mitochondrial shrinkage and cristae fragmentation, were observed in the shPCK2 group (Figure 5G). These results provide morphological evidence supporting the critical role of PCK2 in maintaining mitochondrial function and cellular antioxidant defense in chemoresistant cells.

After overexpressing PCK2 in A549 cells, our results showed that the decrease in GPX4 expression in the DDP treated group was significantly attenuated compared with the control group (Figure S3A), indicating that PCK2 may mediate ferroptosis resistance. Further investigations revealed that PCK2 overexpression in A549 led to an increase in IC_50_ of DDP, suggesting that PCK2 is involved in regulating chemoresistance. Notably, the Erastin‐induced reduction in IC_50_ was partially reversed by PCK2 overexpression (Figure S3B). Additionally, following 48 h of DDP treatment, no significant changes in GSH and MDA levels were observed in OE‐PCK2 cells, whereas marked alterations were detected in the control group (Figure S3C,D). These findings uncover the potential role of PCK2 in regulating antioxidant stress and chemoresistance in NSCLC cells.

### 3.6 RGB‐286638 Free Base Inhibits Cell Proliferation and Binds Tightly to PCK2

**FIGURE 6:**
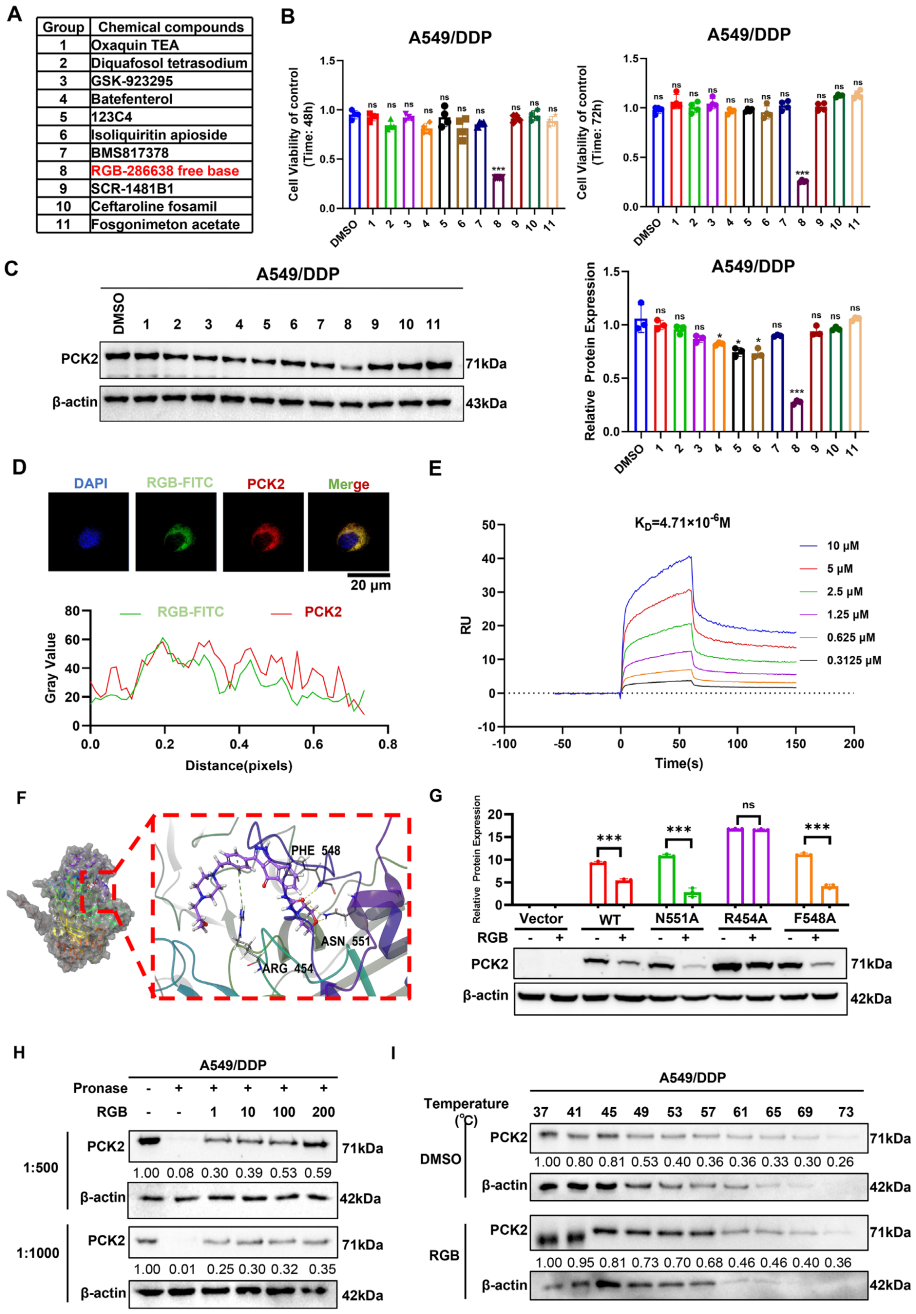
RGB‐286638 free base binds tightly to PCK2 and inhibits A549/DDP cell proliferation. (A) Names of 11 compounds. (B) Cell viability of A549/DDP cells treated with compounds for 48 and 72 h. (C) PCK2 expression in A549/DDP cells treated with compounds for 48 h. (D) Colocalization of RGB and PCK2. (E) SPR of RGB and PCK2. (F) Molecular docking between RGB and PCK2. (G) Site‐directed mutagenesis assay. (H) Drug Affinity Responsive Target Stability assay (DARTS). (I) Thermal Shift Assay (TSA). (ns, no significant, *p < 0.05, ***p < 0.001).

To further enhance the translational significance of PCK2, we treated A549/DDP cells with 11 compounds (Figure 6A) for 48 and 72 h. Among these compounds, RGB exhibited the most potent inhibitory effect on cell proliferation (Figure 6B). Differential analysis revealed 2593 up‐regulated genes (*p*‐value < 0.05, log_2_FoldChange > 2) and 2176 down‐regulated genes (*p‐*value < 0.05, log_2_FoldChange < −2) in A549 cell line treated with RGB (Figure S4A). Up‐regulated genes enriched in pathways related to oxidative stress and also ferroptosis (Figure S4B,C). Meanwhile, we conducted combination experiments using RGB and inhibitors of different types of programmed cell death. The results demonstrated that ferroptosis inhibitors Fer‐1 could effectively reverse RGB‐induced cell death, which further confirmed that ferroptosis is involved in the anti‐tumor effect of RGB on chemoresistant cells (Figure S4D). GSH was significantly decreased in a RGB dose‐dependent manner (0, 0.5, and 1 μM), while the MDA was relatively increased (Figure S4E,F). Compared with the control group, the Lipid ROS in RGB‐treated groups exhibited a significant dose‐dependent elevation (Figure S4G,H). Additionally, FerroOrange probe staining revealed that the intracellular Fe^2+^ content was also increased (Figure S4I).

Concurrently, RGB exerted the strongest inhibitory effect on PCK2 expression (Figure 6C). We performed confocal microscopy using FITC‐labeled RGB, which demonstrated robust colocalization of the two molecules (Figure 6D). SPR assays further confirmed their high binding affinity (Figure 6E). Virtual molecular docking simulations identified three potential binding sites: ASN551, ARG454, and PHE548 (Figure 6F). Site‐directed mutagenesis experiments showed that only mutation of the ARG454 residue abrogated the inhibitory effect of RGB on PCK2 (Figure 6G). Finally, DARTS assay and TSA were employed to verify the binding interaction between RGB and PCK2. In the DARTS assay, the proteolytic resistance of PCK2 was significantly enhanced upon RGB treatment, indicating a direct binding between them (Figure 6H). Meanwhile, the TSA results showed a marked increase in the thermal stability of PCK2 in the presence of RGB, further confirming the high‐affinity binding of RGB to PCK2 (Figure 6I).

### 3.7 RGB‐286638 Free Base Reverses DDP‐Resistance Through Ferroptosis by Inhibiting PCK2

**FIGURE 7:**
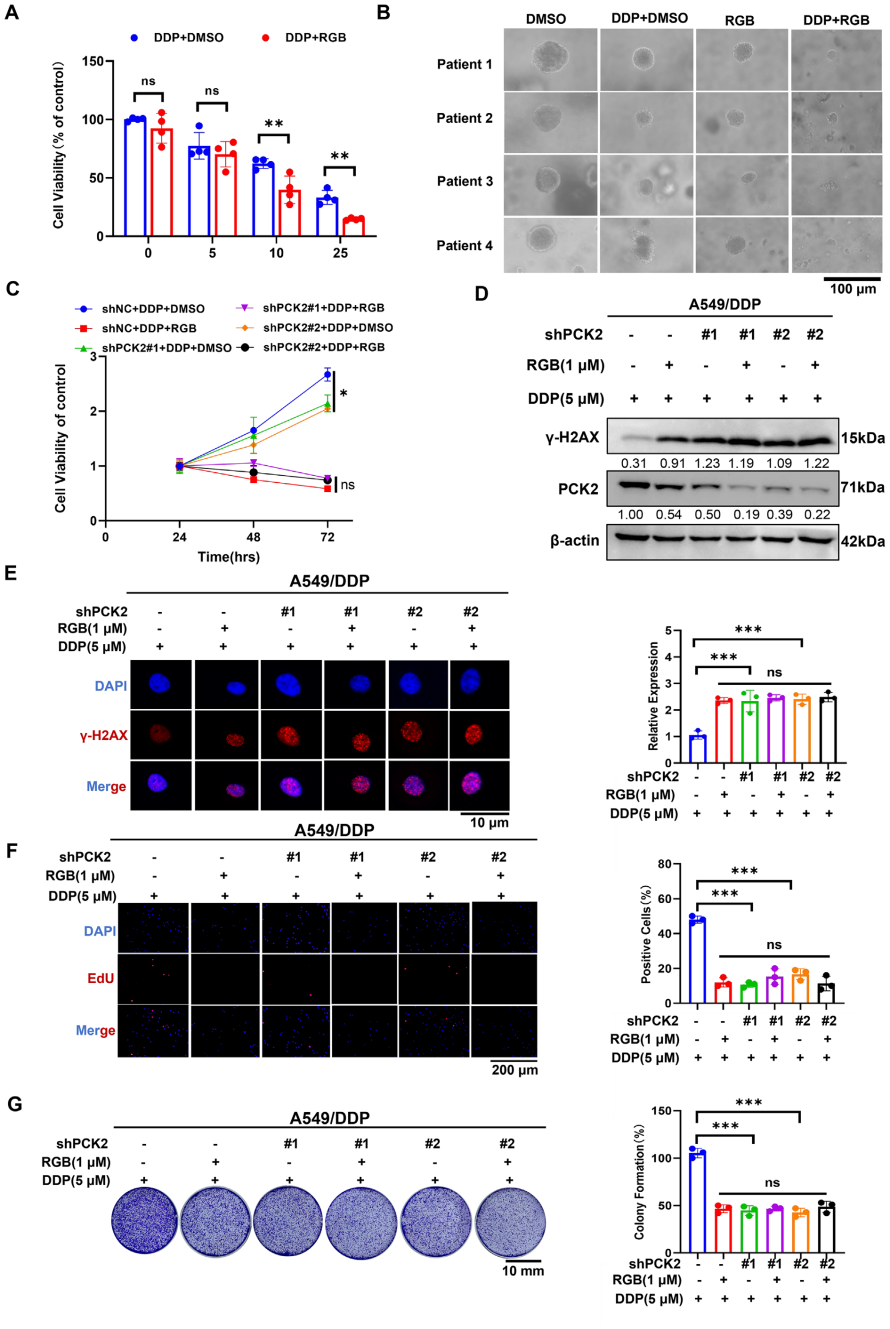
RGB‐286638 free base combined with DDP inhibits proliferation of A549/DDP cells. (A) Cell viability of A549/DDP cells upon combined treatment with RGB and DDP. (B) Morphological changes of organoids upon combined treatment with RGB and DDP. (C) Cell proliferation in A549/DDP shNC and A549/DDP shPCK2 cell lines after 48 h of combined treatment with DDP + DMSO or DDP + RGB. (D) γ‐H2AX expression in A549/DDP shNC and A549/DDP shPCK2 cell lines after 48 h of combined treatment with DDP + DMSO or DDP + RGB. (E) γ‐H2AX immunofluorescence expression. (F) EdU assay. (G) Colony formation assay. (ns, no significant, ***p < 0.001).

Combination treatment with RGB and DDP in chemoresistant cells significantly reduced cell proliferation (Figure 7A) and induced marked morphological changes in organoids, including shrinkage and fragmentation (Figure 7B). In A549/DDP cells, after combined 48 h treatment with RGB and DDP, there was no significant difference in the cell proliferation inhibition rate between A549/DDP shNC and A549/DDP shPCK2 cells (Figure 7C), confirming that RGB affects DDP sensitivity of tumor cells by regulating PCK2. Compared with DDP monotherapy, γ‐H2AX in the DDP + RGB combination group increased significantly, indicating that the combined use of the two drugs could synergistically induce more severe DNA damage. However, in shPCK2 cells, γ‐H2AX in the combined treatment group did not further increase (Figure 7D). Immunofluorescence results were highly consistent with results above (Figure 7E). Through EdU and colony formation assays, we also confirmed that RGB enhances the damaging effect of DDP in chemoresistant cells by inhibiting PCK2 (Figure 7F,G). RGB may enhance the cytotoxic effect of DDP by downregulating PCK2. This finding provides a new potential therapeutic strategy for overcoming DDP resistance and lays a foundation for subsequent studies on the molecular mechanism of PCK2 in tumor drug resistance.

Meanwhile, under the combination of two drugs, we observed an increase in intracellular Lipid ROS and MDA levels, accompanied by a corresponding decrease in GSH (Figure S5A–C). However, after PCK2 knockdown, RGB failed to further enhance ferroptosis in cells (Figure S5A–C). These findings fully demonstrate that RGB modulates the sensitivity of cells to DDP by mediating PCK2.

### 3.8 RGB Activated Ferroptosis in DDP‐Resistant Cells and Restored Their Sensitivity to DDP

**FIGURE 8:**
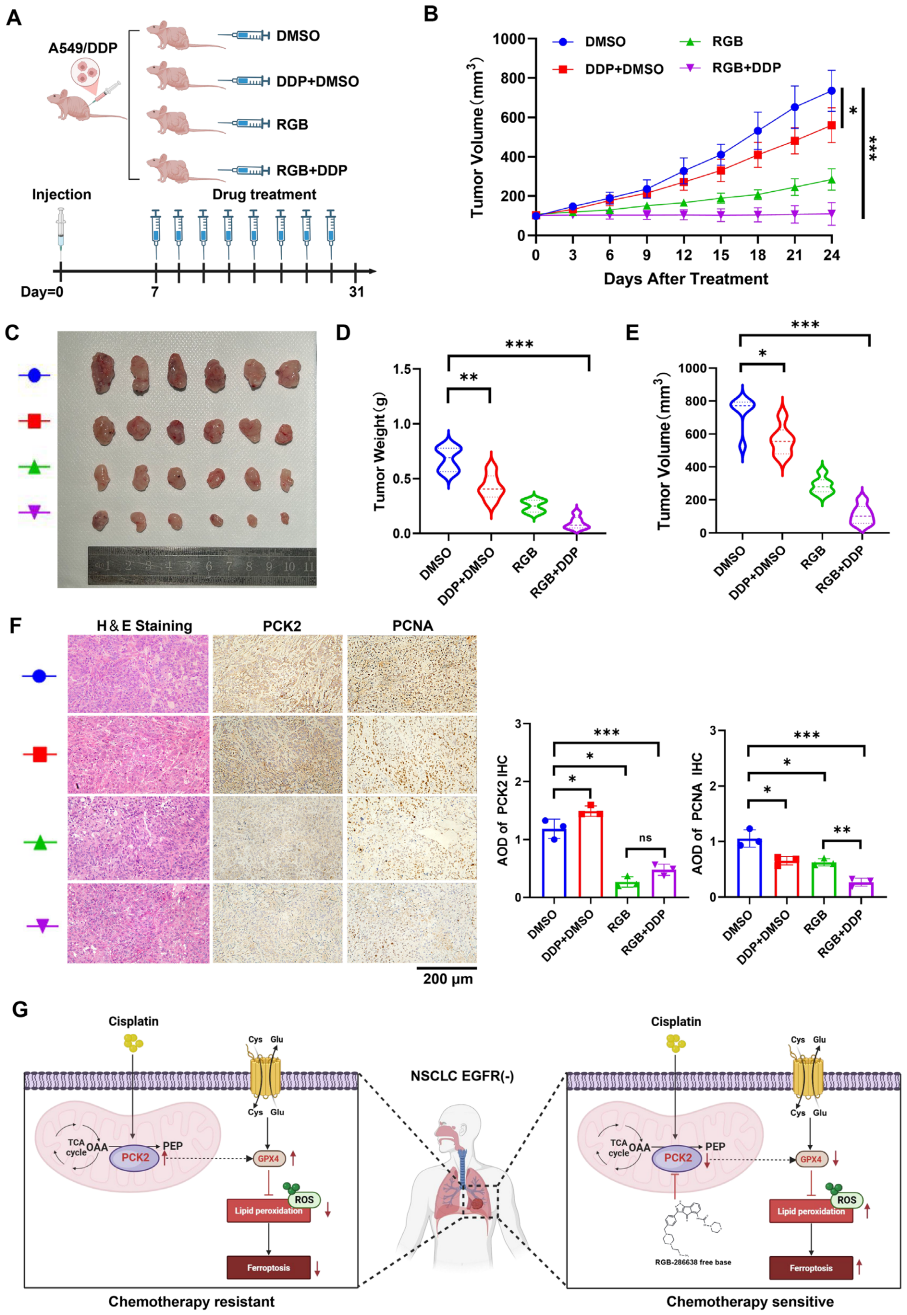
Combination of RGB‐286638 free base and DDP inhibits tumor proliferation in vivo. (A) Experimental scheme: DMSO, DDP (5 mg/kg), RGB (30 mg/kg), DDP + RGB. (B) Tumor growth curve. (C) Tumor morphology. (D) Tumor weight. (E) Tumor volume. (F) HE and IHC of tumor tissues. (G) Schematic diagram. (ns, no significant, *p < 0.05, **p < 0.01, ***p < 0.001).

Nude mice were randomly divided into four groups and inoculated with an equal number of A549/DDP cells (Figure 8A). After tumor formation, the mice were intraperitoneally injected with DMSO, DDP (5 mg/kg), RGB (30 mg/kg), or combination of DDP and RGB, respectively. We found that the tumor volume and weight in the combination group were significantly smaller than those in the DDP monotherapy group (Figure 8B–E). IHC further revealed that the expression of PCNA in tumor tissues was markedly downregulated following the combined treatment with DDP and RGB (Figure 8F). Furthermore, RGB exhibited no significant toxic effects on the heart, liver, spleen, lung, and kidney in vivo (Figure S6). These findings fully demonstrate that RGB can effectively enhance the cytotoxic effect of DDP against drug‐resistant cells. Collectively, PCK2 regulates NSCLC DDP resistance via ferroptosis. Targeting PCK2 with RGB provides a promising strategy to overcome chemoresistance, facilitating improved clinical interventions for NSCLC (Figure 8G).

## 4 Discussion

Lung cancer stands as one of the most prevalent malignancies globally and the foremost contributor to cancer‐associated mortality, with approximately 2 million new cases diagnosed and 1.76 million deaths reported annually [17]. Delayed diagnosis and acquired resistance to current chemotherapy are the primary contributors to the high mortality burden of lung cancer [1, 18]. Although platinum‐based agents remain the cornerstone of clinical treatment for NSCLC, only 20%–30% of patients derive clinical benefit from such therapy, a limitation predominantly driven by the emergence of chemoresistance [4]. Ferroptosis has assumed a critical role in advancing our understanding and management of cancer chemoresistance in clinical and translational settings. Mounting studies have demonstrated that the impaired induction of ferroptosis is a widespread hallmark of chemoresistant tumors, as cancer cells often hijack ferroptosis‐suppressive mechanisms to survive chemotherapy‐induced oxidative damage [19, 20, 21, 22, 23, 24]. Such breakthroughs not only enrich the therapeutic arsenal against recalcitrant cancers but also provide a rational basis for personalized treatment strategies tailored to the ferroptosis status of individual tumors.

In summary, this study systematically explores the intricate crosstalk between ferroptosis and chemoresistance, culminating in the identification of PCK2 as a novel regulatory node. PCK2, a mitochondria‐localized metabolic kinase, exerts a crucial regulatory role in the development of ferroptosis resistance [25]. PCK2 is involved in enhancing ferroptosis suppression, ultimately conferring chemoresistance on NSCLC. Moreover, we successfully developed a novel inhibitor RGB that specifically targets PCK2. This inhibitor not only effectively suppresses the activity of PCK2 but also restores the sensitivity of chemoresistant cancer cells to conventional chemotherapeutic agents by reversing PCK2‐mediated ferroptosis resistance, as validated in both in vitro cell models and in vivo animal models.

The discovery of PCK2 as a ferroptosis‐related chemoresistance target fills the gap in the current understanding of the molecular mechanisms underlying cancer chemoresistance, particularly clarifying the regulatory cascade linking PCK2 to ferroptosis resistance. This finding provides a new theoretical basis for overcoming treatment failure in clinical oncology. Meanwhile, the novel PCK2 inhibitor RGB developed in this study exhibits promising pharmacological properties, including high specificity for PCK2, low systemic toxicity, and favorable bioavailability. These characteristics lay a solid foundation for the translation into clinical practice, offering a potential combinatorial therapeutic strategy for chemoresistant tumors.

Nevertheless, this study has certain limitations. For instance, the detailed molecular mechanisms by which PCK2 regulates ferroptosis resistance—such as the specific downstream signaling molecules and post‐translational modification events involved—require further in‐depth investigation. Future research will focus on addressing these limitations.

Collectively, our findings highlight PCK2 as a promising therapeutic target for reversing chemoresistance by modulating ferroptosis, and the novel inhibitor RGB holds great translational potential for the clinical treatment of chemoresistant cancers.

## Author Contributions

**Jing Liu**, **Ziyan Wang**, and **Chen Gu:** data curation, formal analysis, validation, writing – review and editing. **Yili Chen** and **Yang Yang:** data curation, software. **Jianjie Zhu** and **Yihua Zhang:** formal analysis, methodology, software, validation. **Yuna Shao** and **Xiaodong Pang:** methodology, visualization. **Yujie Ge**, **Huiwen Qian**, and **Jian‐an Huang:** validation, visualization. **Zeyi Liu**, **Yuanyuan Zeng**, and **Cheng Ji:** data curation, conceptualization, funding acquisition, writing – review and editing.

## Funding

This work was supported by grants from the National Natural Science Foundation of China (82272648), Jiangsu Provincial Medical Key Discipline (ZDXK202201), and Clinical Diagnosis and Treatment Technology Innovation Project (CXZL‐F‐240701).

## Ethics Statement

This study was performed in compliance with the Declaration of Helsinki and its subsequent revisions. The human tissue collection and research protocols were approved by the Medical Ethics Committee of Soochow University (Ethics Code SUDA20250827A02) and the First Affiliated Hospital of Soochow University (Ethics Code 2025668). All animal experiments were conducted under protocols approved by the Animal Ethics Committee of Soochow University (Ethics Code 202508A0479).

## Conflicts of Interest

The authors declare no conflicts of interest.

## Supporting information


**Figure S1:** DDP induces ferroptosis resistance in NSCLC cells.
**Figure S2:** Overexpression of PCK2 reduces sensitivity of NSCLC to DDP.
**Figure S3:** PCK2 confers resistance to ferroptosis in NSCLC.
**Figure S4:** RGB‐286638 free base enhances ferroptosis.
**Figure S5:** RGB‐286638 free base reverses DDP‐resistance to ferroptosis by inhibiting PCK2.
**Figure S6:** H&E staining of drug‐induced tissue injury in vivo.

## Data Availability

All datasets utilized and analyzed in this study can be obtained from the corresponding author on reasonable request.
