# Supplementary material for: PCK2 Inhibition Reverses Cisplatin Resistance of Non‐Small Cell Lung Cancer by Triggering Ferroptosis
Source: FASEB J. 2026 Aug 14;40(16):e72195. doi: 10.1096/fj.202602464R (PMC13475953; doi:10.1096/fj.202602464R)
Supplement: Supplementary file 1 [file FSB2-40-e72195-s001.docx]

**Figure S1**

**Figure S1.** **DDP induces ferroptosis resistance in NSCLC cells**

A. GSH in A549 cells after DDP treatment. B. MDA in A549 cells after DDP treatment. C-D. Lipid ROS in A549 cells treated with DDP for 48 h. E. Intracellular Fe²⁺ in A549 cells after DDP treatment. F. Ferroptosis and apoptosis associated protein expression in A549 cells treated with DDP. G. Ferroptosis and apoptosis associated protein expression in H1299 cells treated with DDP. (ns, no significant, **P* < 0.05, ***P* < 0.01, ****P* < 0.001)

**Figure S2**

**Figure S2. Overexpression of PCK2 reduces sensitivity of NSCLC to DDP**

A. Protein expression in A549 OE-PCK2 cells. B. mRNA expression in A549 OE-PCK2 cells. C. Cell viability under treatment DDP. D. Proliferative capacity of cells with EdU. E. Colony formation assay. F. Fluorescence expression of γ-H2AX induced by PCK2 overexpression under DDP treatment. G. Protein level of γ-H2AX after PCK2 overexpression under DDP treatment. (ns, no significant, **P* < 0.05, ***P* < 0.01, ****P* < 0.001)

**Figure S3**

**Figure S3. PCK2 confers resistance to ferroptosis in NSCLC.**

A. Ferroptosis-related protein expression in A549 OE-PCK2 cells following 48 h of DDP treatment. B. IC₅₀ values of Erastin in A549, A549 OE-PCK2 and A549 OE-PCK2 treated with Erastin cells. C. GSH levels in A549 and A549 OE-PCK2 cells treated with DMSO or DDP. D. MDA levels in A549 and A549 OE-PCK2 cells treated with DMSO or DDP. (ns, no significant, **P* < 0.05, ***P* < 0.01, ****P* < 0.001)

**Figure S4**

**Figure S4. RGB-286638 free base Enhances Ferroptosis**

A-C. Pathway enrichment analysis related to RGB. D. Rescue effect of cell death inhibitors on RGB-induced cell death. E. GSH in A549/DDP cells after RGB treatment. F. MDA in A549/DDP cells after RGB treatment. G-H. Lipid ROS in A549/DDP cells treated with RGB for 48 h. I. Intracellular Fe²⁺ in A549/DDP cells after RGB treatment. (**P* < 0.05, ***P* < 0.01, ****P* < 0.001)

**Figure S5**

**Figure S5.** **RGB-286638 free base reverses DDP-resistance to ferroptosis by inhibiting PCK2**

A. Lipid ROS in A549/DDP cells treated with RGB combined with DDP. B. GSH in A549/DDP cells treated with RGB combined with DDP. C. MDA in A549/DDP cells treated with RGB combined with DDP. (ns, no significant, **P* < 0.05, ***P* < 0.01, ****P* < 0.001)

**Figure S6**

**Figure S6. H&E staining of drug-induced tissue injury *in vivo***

H&E staining was used to assess RGB and DDP-induced tissue injury in the heart, liver, spleen, lungs, and kidneys.
